# Explore the Usefulness of Concurrent Chemotherapy in Stage II Nasopharyngeal Carcinoma: A Retrospective Study

**DOI:** 10.3389/fphar.2021.688528

**Published:** 2021-09-21

**Authors:** Pei-Jing Li, Yu-Lin Lai, Fang He, Yuan-Yuan Chen, Zhuo-Sheng Gu, Wei Luo, Qun Zhang

**Affiliations:** ^1^ Department of Radiation Oncology, Cancer Hospital of University of Chinese Academy of Sciences (Zhejiang Cancer Hospital), Zhejiang Key Laboratory of Radiation Oncology, Institute of Cancer Research and Basic Medical Sciences of Chinese Academy of Sciences, Hangzhou, China; ^2^ Department of Radiotherapy, First Affiliated Hospital of Sun Yat-sen University, Guangzhou, China; ^3^ Department of Radiotherapy, Sixth Affiliated Hospital of Sun Yat-sen University, Guangzhou, China; ^4^ Department of Radiotherapy, State Key Laboratory of Oncology in South China, Collaborative Innovation Center for Cancer Medicine, Sun Yat-sen University Cancer Center, Guangzhou, China

**Keywords:** radiotherapy, nasopharyngeal carcinoma, chemotherapy, intensity-modulated radiotherapy (IMRT), two-dimensional radiotherapy (2DRT)

## Abstract

**Objective:** This study aims to compare the treatment outcomes of concurrent chemoradiotherapy (CCRT) versus radiotherapy (RT) alone in stage II nasopharyngeal carcinoma (NPC) patients.

**Methods:** We retrospectively collected 601 stage II NPC patients treated in two hospitals between June 2003 to June 2016. All patients were divided into the CCRT group (*n* = 255) and the RT group (*n* = 346). Overall survival (OS), locoregional failure-free survival (LRFFS), progression-free survival (PFS), and distant metastasis-free survival (DMFS) were assessed using the Kaplan-Meier method. The log-rank test was used to compare the differences between the groups. The Cox-regression hazards model was performed to determine potential prognostic factors.

**Results:** The median follow-up was 99 months. No significant difference was found in locoregional recurrence, distant metastasis, disease progression, and death between the two groups (all *p* > 0.05). In univariate analysis, the 5-years OS, PFS, LRFFS, and DMFS had no significant differences between the CCRT and RT groups (all *p* > 0.05). Two-dimensional radiotherapy (2DRT) sub-analysis showed that CCRT remarkably increased DMFS, PFS, and OS rates (all *p* < 0.05) but not LRFFS (*p* = 0.258) compared with RT alone. While intensity-modulated radiotherapy (IMRT) sub-analysis showed that the prognosis of the two groups had no significant differences (all *p* > 0.05). In multivariate analyses, age was significantly and inversely related to OS, PFS, LRFFS, and DMFS. IMRT was an independent favorable factor for improving LRFFS, PFS, and OS. Concurrent chemotherapy was an independent protective factor for DMFS.

**Conclusion:** In the context of 2DRT, it is definite that concurrent chemotherapy provides survival benefits for patients with stage II NPC. While in the IMRT era, the impact of chemotherapy on survival in patients with stage II NPC is weakened. Prospective randomized controlled studies are required to confirm these results.

## Introduction

Nasopharyngeal carcinoma (NPC) is a distinctive head and neck cancer. It has an extremely skewed geographic distribution. According to Global Cancer Statistics, approximately 129,000 new NPCs were diagnosed worldwide in 2018, and more than 70% of cases were reported in East and Southeast Asia (Bray et al., 2018; Chen et al., 2019). As it is radio-sensitive and chemo-sensitive, radiotherapy combined with chemotherapy is the standard treatment for patients with NPC(Chen et al., 2019; Pfister et al., 2021). With substantial advances in screening and diagnosis, increasingly more early-stage (stage I-II) patients have been diagnosed. In the context of two-dimensional radiotherapy (2DRT), it has been confirmed by a prospective randomized controlled trial (RCT) that concurrent chemoradiotherapy (CCRT) was superior to radiotherapy (RT) alone in the treatment of stage II NPC (Chen et al., 2011). However, 2DRT had gradually been substituted by intensity-modulated radiotherapy (IMRT) in the past 2 decades. The 5-years overall survival (OS) rate of stage II NPC patients has changed from 85% by using 2DRT to 95% using IMRT alone, mainly contributed by enhanced locoregional control rates (Chen et al., 2011; Chen et al., 2016; Su et al., 2016). Simultaneously, it is with more acceptable toxicity and a better quality of life (QOL) for patients with NPC receiving IMRT than 2DRT (Su et al., 2012; Tham et al., 2010; Pan et al., 2017a; Pan et al., 2017b). (Tham et al., 2010; Su et al., 2012; Pan et al., 2017a; Pan et al., 2017b). In facing the striking therapeutic effect of IMRT in early-stage NPC patients, many oncologists have considered omitting chemotherapy for stage II patients in the IMRT era. Nevertheless, there is an absence of robust evidence-based recommendations in managing stage II NPC. The treatment of stage II NPC remains controversial, and the actual benefits of chemotherapy in these patients are unclear. Therefore, this study aims to assess the treatment outcomes of CCRT versus RT alone in treating patients with stage II NPC.

## Patients and Methods

### Patients

This study retrospectively integrated clinic data of newly diagnosed stage II NPC patients from the Cancer Hospital of the University of Chinese Academy of Sciences (Zhejiang Cancer Hospital) and Sun Yat-Sen University Cancer Center. All patients received definitive treatment between June 2003 to June 2016. Inclusion criteria were: (1) 18–75 years old; (2) pathologically diagnosed as stage II NPC (restaged according to the 7th edition of the AJCC/UICC staging system); (3) completion of radical radiation. The exclusion criteria were: (1) previous treatment of NPC; (2) patients who had a secondary malignancy. This study had approval from the institutional review board (IRB-2021–90), and the requirement for informed consent was waived.

### Treatments and Follow-Up

All patients received RT alone (*n* = 346) or CCRT (*n* = 255). Irradiation fields or target volumes were defined according to the tumor extension evaluated by magnetic resonance imaging (MRI). For the 2DRT, the accumulated radiation dose to the primary tumor, lymph node-positive, and lymph node-negative neck tissues was 66–70 Gy, 60–62 Gy, and 50 Gy, respectively. RT was given five times a week at 2 Gy per day. The detailed protocol of 2DRT was the same as the previous study in Guangzhou, China (Chen et al., 2011). For the IMRT, simultaneous modulated accelerated radiation therapy technology was used. The radiation dose was 2.12 Gy or 2.26 Gy per fraction, five fractions per week, a total dose of 66–70 Gy in 30–33 fractions for primary tumor and metastatic lymph node. The detailed IMRT plan was the same as previous studies (Sun et al., 2014; Sun et al., 2016). Concurrent chemotherapy regimens were cisplatin or nedaplatin, 35 mg/ m^2^ weekly (3-6 cycles) or 80–100 mg/ m^2^ every 3 weeks (2-3 cycles). The methods we used to track and monitor patients were described in our previous research (Li et al., 2018). The last follow-up time was July 31, 2020.

### Statistical Analysis

Treatment outcomes were as follows: overall survival (OS), cancer-specific survival (CSS), progression-free survival (PFS), locoregional failure-free survival (LRFFS), and distant metastasis-free survival (DMFS), which were defined as the interval from the onset of radiotherapy to the date of death for any reason, death caused by NPC related events, disease progression, relapse, and distant metastasis, respectively. If an event was absent, the interval was defined from the onset of radiotherapy to the most recent follow-up date. Kaplan-Meier method was conducted in the analysis of the time-to-event endpoints. A Log-rank test was performed in comparison of the differences between the groups. Hazard ratios (HRs) and 95% confidence intervals (CIs) were calculated using Cox regression. Multivariate analyses were used to identify predictive factors for the above endpoints. Comparison of categorical and continuous variables was conducted using the Pearson’s χ2 test and t-test. R software (R version 4.0.2, readr, dplyr, Tableone, VennDiagram, gplots, survival, forestplot, survminer, ggplot2) was used for data analysis. *p* < 0.05 was considered statistically significant.

## Results

### Patient Characteristics

The median follow-up of 500 survivors and all patients was 103 months (m) (range: 17–180 m) and 99 m (range: 15–180 m), respectively. Median survival has not yet been reached. The male-to-female ratio was 2.64:1. Patients with N1 disease were more likely to receive concurrent chemotherapy than those with N0 in the 2DRT era (84.9 *vs.* 70.4%, *p* < 0.05). It has the same trend in the whole group analysis. Details of baseline characteristics and chemotherapy information were summarized in Table 1.

**TABLE 1:** Baseline characteristics.

Evaluate-population	Characteristics		RT group	CCRT group	*p* Value
Whole group			*n* = 346	*n* = 255
	Age (mean ± SD)		47.45 ± 11.09	46.65 ± 10.5	0.373
	Sex (%)	Female	98 (28.3)	67 (26.3)	0.643
		Male	248 (71.7)	188 (73.7)	
	Stage (%)	T1N1	111 (32.1)	94 (36.9)	0.001
		T2N0	110 (31.8)	47 (18.4)	
		T2N1	125 (36.1)	114 (44.7)	
	RT (%)	2DRT	189 (54.6)	159 (62.4)	0.070
		IMRT	157 (45.4)	96 (37.6)	
	CRT (QW)	3–4	−	6 (2.4)	−
		5–6	−	85 (33.3)	
	CRT (Q3W)	1	−	8 (3.1)	
		2–3	−	156 (61.2)	
2DRT subgroup			*n* = 189	*n* = 159	
	Age (mean ± SD)		47.93 ± 10.9	46.27 ± 10.4	0.148
	Sex (%)	Female	55 (29.1)	41 (25.8)	0.570
		Male	134 (70.9)	118 (74.2)	
	Stage (%)	T1N1	77 (40.7)	73 (45.9)	0.005
		T2N0	56 (29.6)	24 (15.1)	
		T2N1	56 (29.6)	62 (39.0)	
	CRT (QW)	3–4	−	4 (2.5)	−
		5–6	−	60 (37.7)	
	CRT (Q3W)	1	−	5 (3.1)	
		2–3	−	90 (56.6)	
IMRT subgroup			*n* = 157	*n* = 96	
	Age (mean ± SD)		46.87 ± 11.1	47.29 ± 10.8	0.765
	Sex (%)	Female	43 (27.4)	26 (27.1)	1.000
		Male	114 (72.6)	70 (72.9)	
	Stage (%)	T1N1	34 (21.7)	21 (21.9)	0.180
		T2N0	54 (34.4)	23 (24.0)	
		T2N1	69 (43.9)	52 (54.2)	
	CRT (QW)	3–4	−	2 (2.1)	−
		5–6	−	25 (26.0)	
	CRT (Q3W)	1	−	3 (3.1)	
		2–3	−	66 (68.8)	

CCRT, concurrent chemoradiotherapy; CRT, concurrent chemotherapy; 2DRT, two-dimensional radiotherapy; IMRT, intensity-modulated radiotherapy; QW, weekly; Q3W, every 3 weeks; RT, radiation; SD, standard deviation.

### Failure Patterns

**FIGURE 1:**
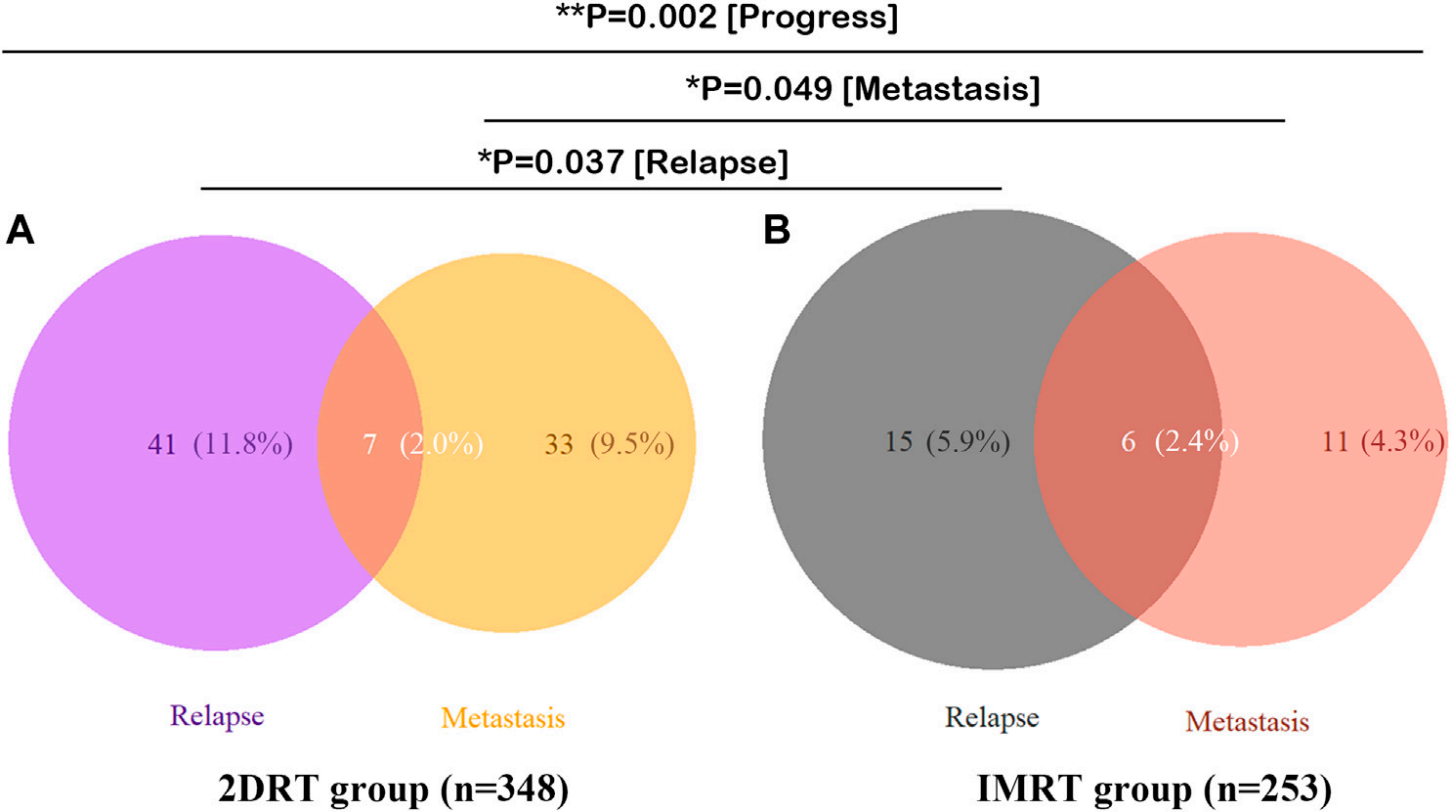
Failure patterns of 601 patients with stage II NPC. **(A)** 2DRT subgroup, **(B)** IMRT subgroup. Abbreviation: 2DRT = two-dimensional radiotherapy, IMRT = intensity-modulated radiotherapy.

Overall, 101 deaths were identified up to the last follow-up, of which 66 (19.1%) happened in the RT group while 35 (13.7%) in the CCRT group (*p* = 0.083). Ninety-one died of the disease, 59 (17.1%) in the RT versus 32 (12.5%) in the CCRT group (*p* = 0.128). And no significant differences were found in term of locoregional recurrence, distant metastasis and disease progression between the RT and CCRT groups in the analysis of whole group level (11.8 *vs.* 11.0%, *p* = 0.741; 11.3% *vs.* 7,1%, *p* = 0.081; 21.4 *vs.* 16.5%, *p* = 0.131, respectively). However, IMRT significantly decreased locoregional recurrence events (*p* = 0.037) and distant metastasis (*p* = 0.049) compared with 2DRT, as was shown in Figure 1.

### Survival Outcomes

**FIGURE 2:**
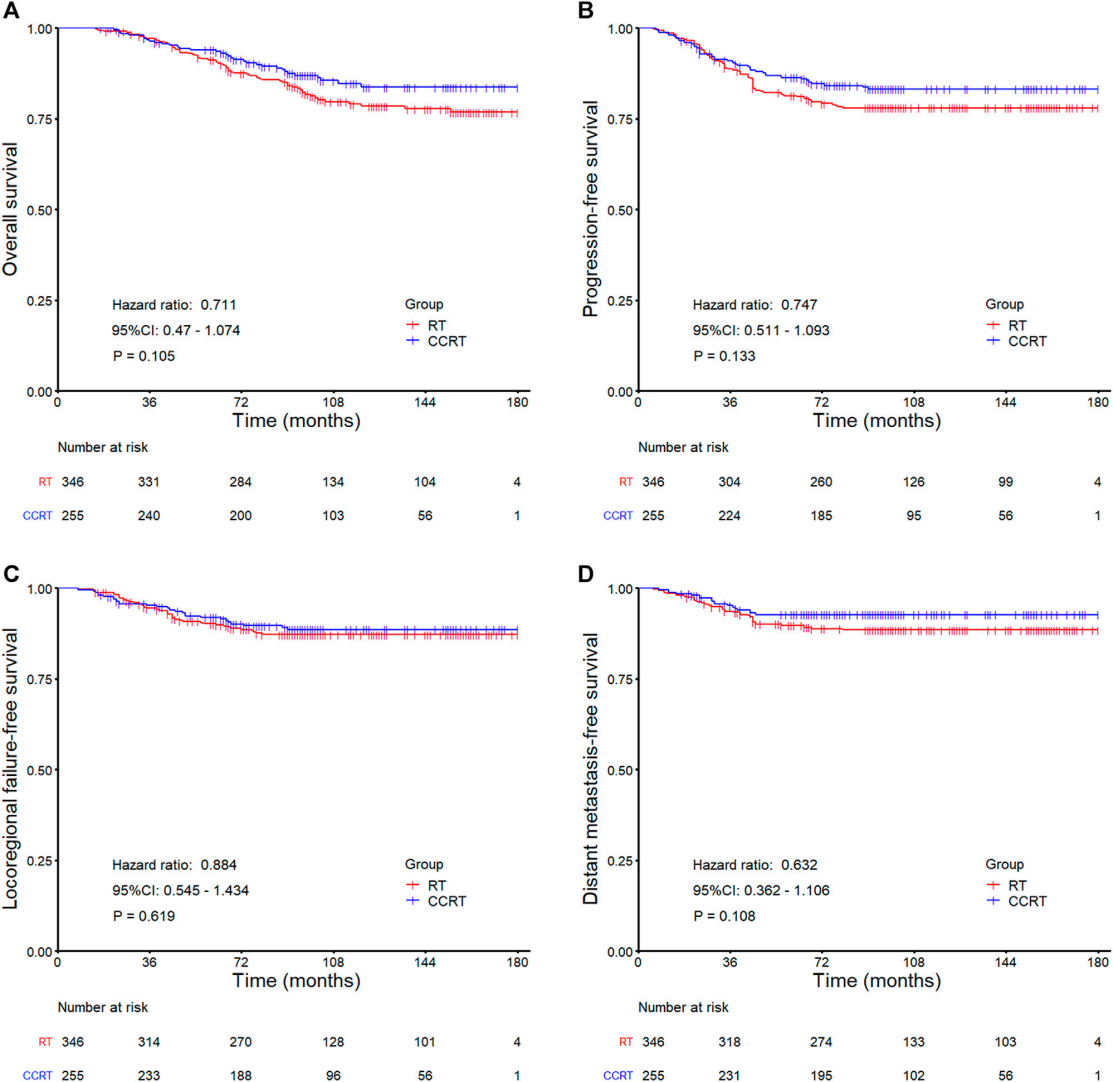
Kaplan-Meier survival curves for the RT and CCRT groups in the whole group: **(A)** Overall survival, **(B)** Progression-free survival, **(C)** Locoregional failure-free survival, **(D)** Distant metastasis-free survival. Abbreviation: RT = radiotherapy, CCRT = concurrent chemoradiotherapy.

**FIGURE 3:**
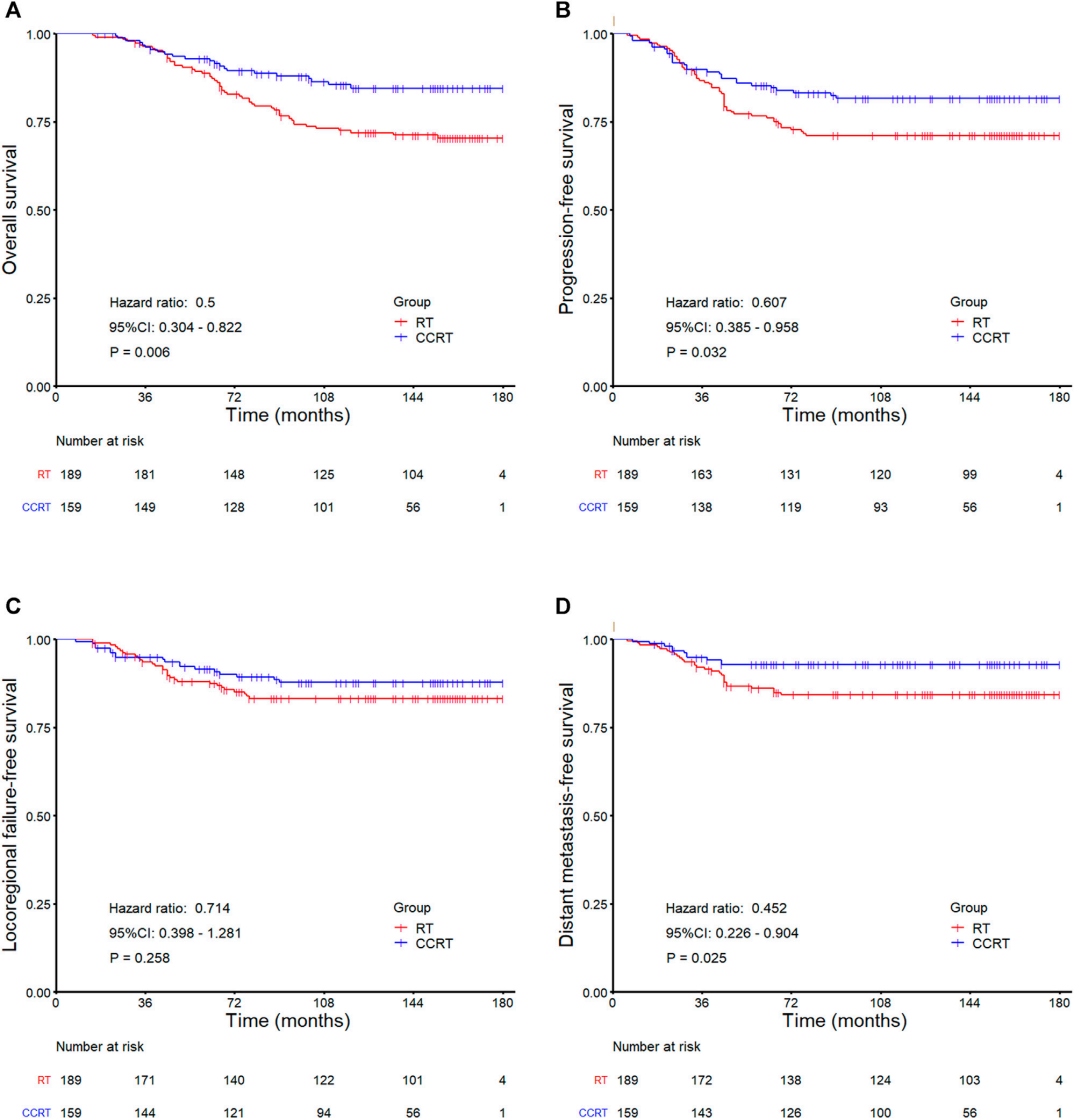
Kaplan-Meier survival curves for the RT and CCRT groups in the 2DRT subgroup: **(A)** Overall survival, **(B)** Progression-free survival, **(C)** Locoregional failure-free survival, **(D)** Distant metastasis-free survival. Abbreviation: RT = radiotherapy, CCRT = concurrent chemoradiotherapy, 2DRT = two-dimensional radiotherapy.

**FIGURE 4:**
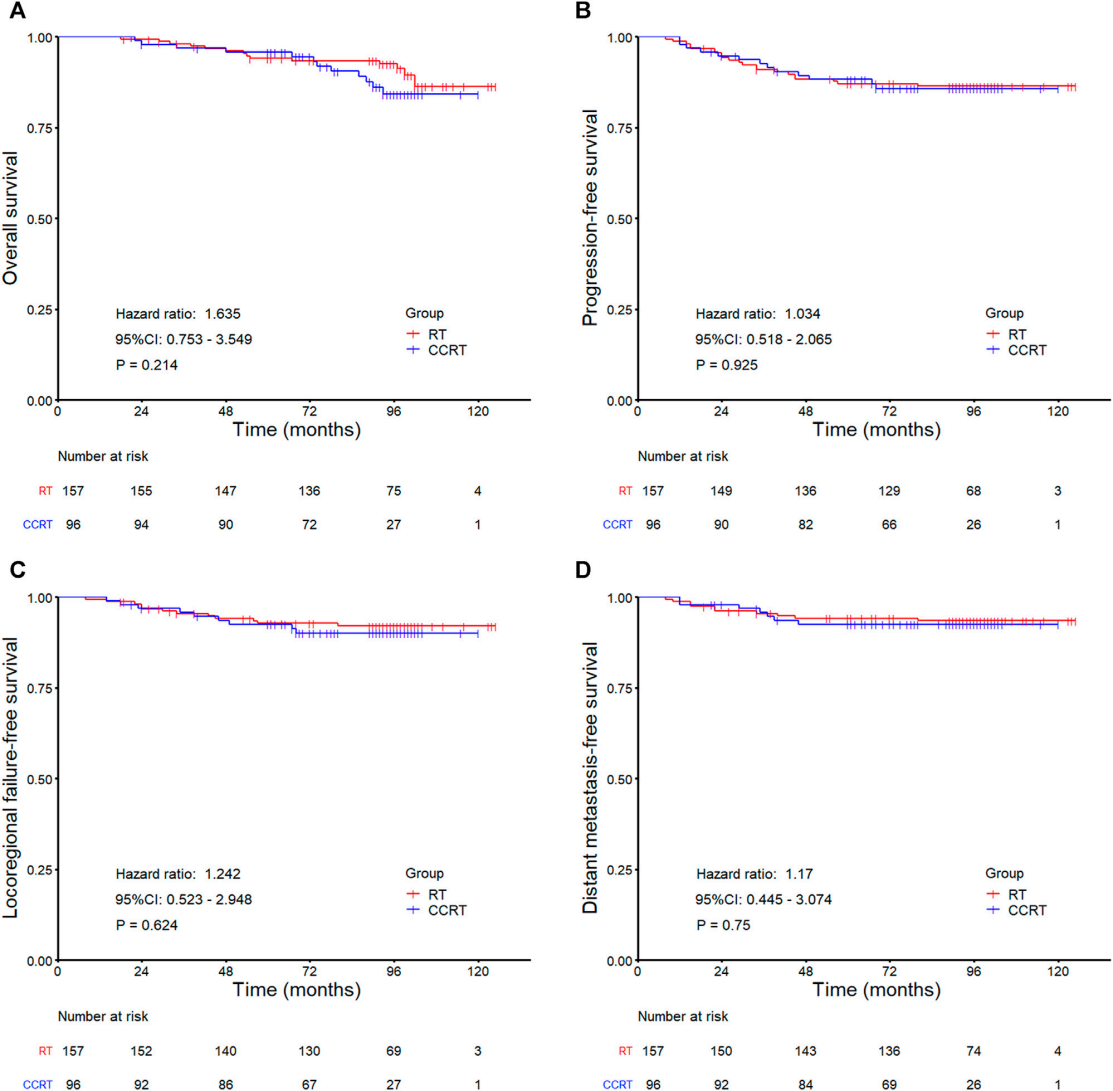
Kaplan-Meier survival curves for the RT and CCRT groups in the IMRT subgroup analysis: **(A)** Overall survival, **(B)** Progression-free survival, **(C)** Locoregional failure-free survival, **(D)** Distant metastasis-free survival. Abbreviation: RT = radiotherapy, CCRT = concurrent chemoradiotherapy, IMRT = intensity-modulated radiotherapy.

In the whole group analyses, the 5-years OS, PFS, LRFFS and DMFS for the CCRT group were similar to that of RT group (91.2 *vs.* 94.0%, *p* = 0.105; 81.7 *vs.* 86.0%, *p* = 0.133; 90.5 *vs.* 91.5%, *p* = 0.619; 89.7 *vs.* 92.7%, *p* = 0.108), along with the 10-years outcomes were shown in Figure 2; Table 2. Then we further did subgroup analysis in 2DRT and IMRT treatment background. Three hundred and forty-eight patients received 2DRT, concurrent chemotherapy could remarkably improve DMFS (HR = 0.452, 95%CI: 0.226–0.904, *p* = 0.025), PFS (HR = 0.607, 95%CI: 0.385–0.958, *p* = 0.032), and OS (HR = 0.500, 95%CI: 0.304–0.822, *p* = 0.006) but not LRFFS (*p* = 0.258), as was shown in Figure 3; Table 2. Two hundred and fifty-three patients treated with IMRT, the prognosis of the RT and CCRT groups had no significant differences (all *p* > 0.05), as was shown in Figure 4 and Table 2. Considering aging-associated disease or death was an issue for long-time follow-up analysis, we added CSS to feature cancer-specific events. As for CSS, similar trends with OS were observed in whole group analysis and subgroup analysis, as shown in (Supplementary Figure S1). To further understand the effect of chemotherapy on different stages of disease under the background of different radiotherapy techniques, we divided the patients into T1-2N1 and T2N0 populations. The results showed that chemotherapy did not bring any survival benefit to T2N0 NPC patients who received 2DRT (Supplementary Figure S2). But it significantly increased DMFS (*p* = 0.012), PFS (*p* = 0.009), and OS (*p* = 0.001) rates of patients with T1-2N1 disease (Supplementary Figure S2). However, in the context of IMRT, chemotherapy could not improve the prognosis either in the T2N0 (Supplementary Figure S3) or T1-2N1 (Supplementary Figure S3) population (all *p* > 0.05).

**TABLE 2:** Comparison of survival outcomes between the RT and CCRT groups in three analysis levels.

Outcomes	Survival rate								
	Whole group			2DRT subgroup			IMRT subgroup		
	RT *n* = 346 (%)	CCRT *n* = 255 (%)	*p* Value	RT *n* = 189 (%)	CCRT *n* = 159 (%)	*p* Value	RT *n* = 157 (%)	CCRT *n* = 96 (%)	*p* Value
OS (y)									
5	91.2	94.0	0.105	88.8	92.9	0.006	94.1	95.8	0.214
10	78.8	83.2		72.5	83.8		86.4	84.2	
CSS (y)									
5	91.2	94.0	0.142	88.8	92.9	0.013	94.1	95.8	0.328
10	81.6	84.7		74.3	84.5		91.3	86.7	
PFS (y)									
5	81.7	86.0	0.133	77.2	84.6	0.032	87.1	88.3	0.925
10	78.3	82.8		71.6	81.0		86.4	85.8	
LRFFS (y)									
5	90.5	91.5	0.619	88.6	90.9	0.258	92.8	92.5	0.624
10	87.5	88.1		83.8	87.1		92.0	90.1	
DMFS (y)									
5	89.7	92.7	0.108	86.1	92.8	0.025	94.2	92.5	0.750
10	88.5	92.7		84.3	92.8		93.5	92.5	

CCRT, concurrent chemoradiotherapy; CSS, cancer-specific survival; DMFS, distant metastasis-free survival; 2DRT, two-dimensional radiotherapy; IMRT, intensity-modulated radiotherapy; LRFFS, locoregional failure-free survival; OS, verall survival; PFS, progression-free survival; RT, radiotherapy.

### Multivariate Analysis

**FIGURE 5:**
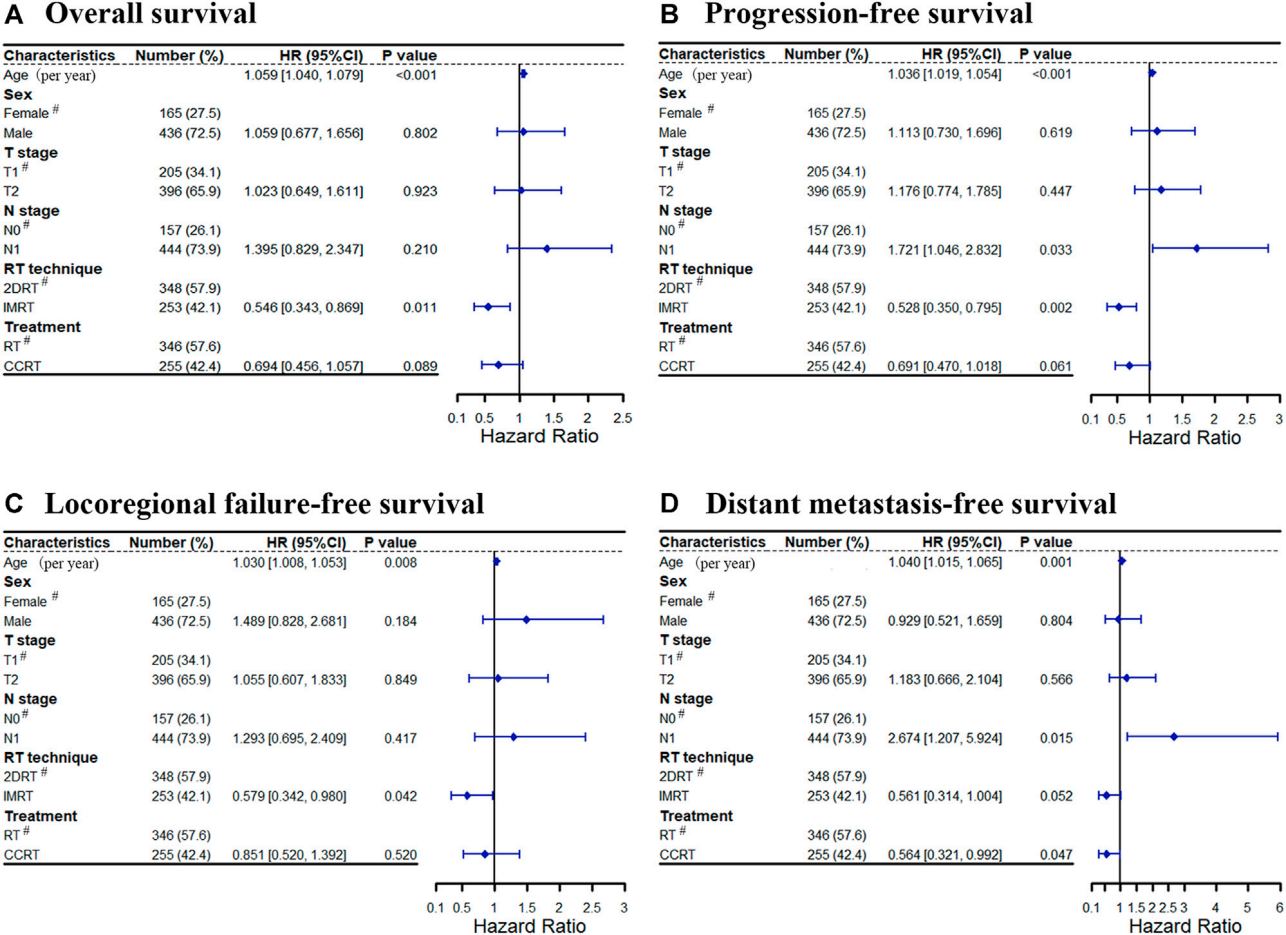
Cox forest for treatment outcomes: **(A)** Overall survival, **(B)** Progression-free survival, **(C)** Locoregional failure-free survival, **(D)** Distant metastasis-free survival. Abbreviation: RT = radiotherapy, CCRT = concurrent chemoradiotherapy, 2DRT = two-dimensional radiotherapy, IMRT = intensity-modulated radiotherapy, HR = hazard ratio, # = reference.

In multivariate analyses, age was an independent prognostic factor for OS (HR = 1.059, 95%CI: 1.040–1.079, *p* < 0.001), PFS (HR = 1.036, 95%CI: 1.019–1.054, *p* < 0.001), LRFFS (HR = 1.030, 95%CI: 1.008–1.053, *p* = 0.008), and DMFS (HR = 1.040, 95%CI: 1.015–1.065, *p* = 0.001). These outcomes were inversely related to age. Then, the RT technique was an independent prognostic factor for LRFFS (HR = 0.579, 95%CI: 0.342–0.980, *p* = 0.042), PFS (HR = 0.528, 95%CI: 0.350–0.795, *p* = 0.002) and OS (HR = 0.546, 95%CI: 0.343–0.869, *p* = 0.011) and tended to affect DMFS (HR = 0.561, 95%CI: 0.341–1.004, *p* = 0.052). IMRT significantly improved the LRFFS, PFS and OS for patients with stage II NPC. In addition, patients with N1 disease had a significantly higher risk of distant metastasis (HR = 2.674, 95%CI: 1.207–5.924, *p* = 0.015) and disease progression (HR = 1.721, 95%CI: 1.046–2.832, *p* = 0.033) than N0 patients. Lastly, concurrent chemotherapy was a significantly favorable prognostic factor for DMFS (HR = 0.564, 95%CI: 0.321–0.992, *p* = 0.047) and tended to reduce the disease progression (HR = 0.691, 95%CI: 0.470–1.018, *p* = 0.061) and mortality (HR = 0.694, 95%CI: 0.456–1.057, *p* = 0.089, respectively). All data were shown in Figure 5.

## Discussion

This retrospective study showed that concurrent chemotherapy could significantly improve treatment outcomes of stage II NPC patients under the background of 2DRT. However, the impact of chemotherapy on survival in the same population was weakened while using IMRT.

Radiotherapy has been established as the primary treatment modality for NPC since 1965. The 5-years OS rate has increased from 50% (1954–1992) to 77% (1990–1999), and then to 85% (2000–2010) (Sun et al., 2019). IMRT is a more advanced technology than 2DRT and has been prevalent since the 1990s. We consider that the gradually increased performance of IMRT contributes to the improvement of locoregional control. It delivers a conformal target with a more uniform dose distribution. IMRT makes it possible to enhance the dose in the target area while strictly restricting the dose to the surrounding organs at risk. Thus, it improves the therapeutic gain ratio. Zhang et al. (2015a) and Lai et al. (2011) conducted large sample retrospective studies. They found that IMRT remarkably improved treatment outcomes of NPC patients in comparison with 2DRT, mainly benefitting from the increased local control rate, especially in early-stage T disease. The present study similarly revealed that IMRT significantly improved local control. Besides, our results displayed that IMRT had a tendency to reduce distant metastasis (HR = 0.561, 95%CI: 0.314–1.004, *p* = 0.052) when adjusted with other potential prognostic factors.

In terms of the 2DRT era, our results favored CCRT for patients with stage II NPC, especially those with regional lymph node metastasis. Two previous researches with high-quality data confirmed that RT combined with chemotherapy significantly improved OS and reduced the risk of distant metastases of patients with stage II NPC in comparison with 2DRT alone. Chua et al. did a post hoc analysis basing on data of two phase III trials. The subgroup analysis indicated that the 5-years OS and DMFS rate was 79 *vs.* 67% (*p* = 0.048) and 86 vs. 74% (*p* = 0.005) in the induction chemotherapy plus RT group and 2DRT alone group, respectively (Chua et al., 2006). Chen et al. (2011), Li et al. (2019) conducted a RCT and demonstrated that concurrent chemotherapy increased 5-years OS by 10% (94.5 vs 85.8%, *p* = 0.007) and 10-years OS by 18% (83.6 vs. 65.8%, *p* = 0.001) in stage II NPC patients. Ten years follow-up of Chen’s study demonstrated that chemotherapy mainly played a role in T2N1 NPC patients. This was consistent with our findings.

Under the background of IMRT, several retrospective studies and meta-analyses deemed that chemotherapy provided no survival benefit in treating patients with stage II NPC. In other words, these studies believed that IMRT alone was sufficient for this population (Zhang et al., 2015b; Chen et al., 2016; Su et al., 2016; Pan et al., 2017c; Xu et al., 2017; Wang et al., 2018; Liu et al., 2018; Chen et al., 2018; Liu et al., 2018)–(Zhang et al., 2015b; Chen et al., 2016; Su et al., 2016; Pan et al., 2017c; Xu et al., 2017; Wang et al., 2018; Liu et al., 2018; Chen et al., 2018; Liu et al., 2018). A meta-analysis by Liu et al. (2018) reviewed seven studies of 1,302 stage II NPC patients who received IMRT. Their results showed that IMRT plus concurrent chemotherapy had no improvement in prognosis comparing with IMRT alone. But CCRT notably increased the risk of acute grade 3–4 hematological toxicity. Considering the excellent results achieved by IMRT, many scholars thought it was overtreated by adding chemotherapy and recommended that chemotherapy may not be necessary for stage II NPC patients treated with IMRT. However, only one study was prospectively conducted among the seven studies included in Liu’s meta-analysis but with small sample size. The result is not convincing enough. In addition, there are many studies with conflicting results. A series of studies indicated that stage II NPC patients who received IMRT alone had worse treatment outcomes than those who underwent chemoradiotherapy (Luo et al., 2014; Guo et al., 2016; Ahmed et al., 2019; He et al., 2019)–(Luo et al., 2014; Guo et al., 2016; Ahmed et al., 2019; He et al., 2019). A study by Guo et al. (2016) showed that the addition of chemotherapy could improve LRFFS (HR: 0.263, 95% CI 0.083–0.839, *p* = 0.024) in stage II NPC patients, especially for T1N1 disease. Zong et al. (2015) reported a 5-years accumulated distant metastasis rate of 10.8% in patients with T1-2N1 disease versus 0.1% in patients with T1-2N0 NPC, accompanied by significantly different OS rates of 84.7 *vs.* 95.4% (*p* = 0.005). Thus, some researchers considered N-positive NPC patients as a unique subgroup in the IMRT era. Treatment outcomes were far from satisfactory. It may be inappropriate to remove chemotherapy in this group of patients because the toxicities associated with salvage treatments for recurrent disease after RT alone may be greater than those related to chemotherapy. Up to now, there have been only two prospective trials focusing on chemoradiotherapy in the literature. Chen et al. (2018) compared the efficacy of CCRT + adjuvant chemotherapy (AC) (*n* = 81) with IMRT alone (*n* = 79) in treating stage II NPC patients (AJCC 7th edition). They gave a preliminary report that CCRT + AC did not achieve more favorable 5-years OS, LRFFS, and DMFS rates than IMRT alone (OS: 91.4 vs. 88.6%, LRFFS: 96.26 vs. 93.67%, DMFS: 93.82 vs. 93.67%, all *p* > 0.05, with a median follow-up of 61.5 m). Another phase II clinical study (Huang et al., 2020) evaluated the efficacy of concurrent chemotherapy versus IMRT alone. Eighty-four stage II NPC (AJCC 7th edition) were recruited, all of whom received IMRT alone (*n* = 43) or CCRT (*n* = 41). The OS, local failure-free survival (LFFS), regional failure-free survival (RFFS), and DMFS for the CCRT group and IMRT alone group were 100 *vs.* 94.0%, 93.0 vs. 89.3%, 97.7 *vs.* 95.1% and 95.2 *vs.* 94.5%, respectively (all *p* > 0.05). These two trials indicated that chemotherapy yielded no benefit but remarkably increased treatment-associated acute toxicities in stage II NPC patients. Interestingly, these studies both represented that most locoregional recurrence and distant metastases occurred in the T2N1 group, though no statistical differences were found. The study’s author thought it might be due to the small sample size and few events in these two studies. Moreover, the former trial focused on adjuvant chemotherapy. And the sample size of the latter one might not be large enough to possess the power to illustrate the statistical difference. The optimal management for stage II NPC remains controversial. Therefore, we conducted this study to explore the effectiveness of concurrent chemotherapy further. Univariate analysis showed that concurrent chemotherapy provided no survival benefit for stage II NPC patients in whole group analysis. However, multivariate analysis revealed that concurrent chemotherapy was an independent protective factor for improving DMFS after adjusting with other potential prognostic factors (age, sex, stage, and RT technique). That’s not surprising. Multivariate analysis also showed that N1 was a risk factor for distant metastasis. It was consistent with the results of many previous studies (Chua et al., 2003; Xiao et al., 2009; Zong et al., 2005)–(Chua et al., 2003; Zong et al., 2005; Xiao et al., 2009). There were significantly more patients with N1 disease distributed in the CCRT group. In univariate analysis, the actual effect of concurrent chemotherapy might be obscured by the N1 factor. Moreover, it was thought that the effect of concurrent chemotherapy was to increase radiation sensitivity, so that improve local control. In this study, stage II NPC patients who received RT alone achieved an equivalent LRFFS to the CCRT group but underwent a higher distant failure rate than the CCRT arm. On the one hand, as mentioned in Chen’s study, an early-stage disease might have a smaller distant tumor bulk that was more easily eradicated by concurrent chemotherapy (Chen et al., 2011). On the other hand, RT alone was enough for gaining satisfactory local control in early-stage NPC patients. Even though no OS benefit was found by adding concurrent chemotherapy in the era of IMRT, it should be cautious about removing chemotherapy in patients with stage II NPC, especially in patients with regional lymph node metastasis. Potential risk factors like size of metastatic lymph nodes, extracapsular invasion, and the level of EBV DNA should be comprehensively considered when making a treatment strategy for this group of patients.

Results in this study and in the literature showed that the role of concurrent chemotherapy was different in 2DRT and IMRT. The following possible reasons might explain it. Firstly, due to toxicity limitation, the radiation dose to regionally metastatic lymph nodes was higher by using IMRT (66–70 Gy) than 2DRT (60–62 Gy). IMRT significantly improved the locoregional control and even tended to reduce distant metastasis events compared with 2DRT. This narrows the space for chemotherapy to work. Besides, it’s noteworthy that relative lack of precise imaging modalities (e.g., MRI or PET-CT) in the 2DRT era might result in a portion of patients with undetected, more advanced disease being mixed up in the included stage II population. This might contribute to the exaggerated effects (to a certain extent) of chemotherapy in the 2DRT era. Lastly, with the advancement of imaging technology, target delineation is more precise in the IMRT era.

As a retrospective study, there are several points that can’t be ignored. On the one hand, with the advent of various drugs, such as EGRF inhibitors, angiogenesis inhibitors, immune checkpoint inhibitors, etc. and improvements in salvage surgery and re-radiotherapy, patients with relapsed or metastatic disease can continue achieving long-term survival after disease progression. On the other hand, although all patients in this study were staged II NPC, oncologists are prone to give chemotherapy to patients with high-risk factors like bulky tumor volumes, extracapsular invasion, high EBV DNA copy number, etc. Therefore, the results of this study need to be further confirmed by prospective randomized clinical trials with a large sample size. Furthermore, in recent decades, plasma/serum EBV DNA has become an effective prognostic biomarker (Leung et al., 2003; Lin et al., 2004). It complements the TNM staging system for selecting patients at a high risk of developing distant metastasis. Regrettably, EBV DNA data are lacking in the present study owing to incomplete data. Lastly, it is difficult to collect image features that are potentially related to survival and treatment in numeric form and include them in statistical analysis. Therefore, future studies should be designed to incorporate biomarkers such as plasma/serum EBV DNA levels and/or include image features through machine learning to complement the TNM staging system for risk stratification. Overall, our report is noteworthy because of the large population, long-term follow-up, and adoption of multivariate and subgroup analyses. Several Phase II-III trials (NCT02610010, NCT02116231, and NCT02633202) aiming to evaluate the role of CCRT in stage II NPC patients treated with IMRT are ongoing. We are looking forward to their outcomes.

## Conclusion

In the context of 2DRT, it is definite that concurrent chemotherapy provides survival benefits for patients with stage II NPC. While in the IMRT era, the impact of chemotherapy on survival in this population is weakened. Applying a uniform treatment strategy to all the patients in Stage II is inappropriate. Multivariate predictive models and further screening subgroups that suit specific treatment will be a hotspot in future studies.

## Data Availability

The original contributions presented in the study are included in the article/Supplementary Material, further inquiries can be directed to the corresponding author.
