# Supplementary material for: Explore the Usefulness of Concurrent Chemotherapy in Stage II Nasopharyngeal Carcinoma: A Retrospective Study
Source: Front Pharmacol. 2021 Sep 21;12:688528. doi: 10.3389/fphar.2021.688528 (PMC8490628; doi:10.3389/fphar.2021.688528)

Figure S1. Kaplan-Meier survival curves of CSS for the RT and CCRT groups: (a) the whole group, (b) the 2DRT subgroup, (c) the IMRT subgroup. Abbreviation: RT=radiotherapy, CCRT=concurrent chemoradiotherapy, 2DRT=two-dimensional radiotherapy, IMRT=intensity-modulated radiotherapy, CSS=cancer-specific survival.

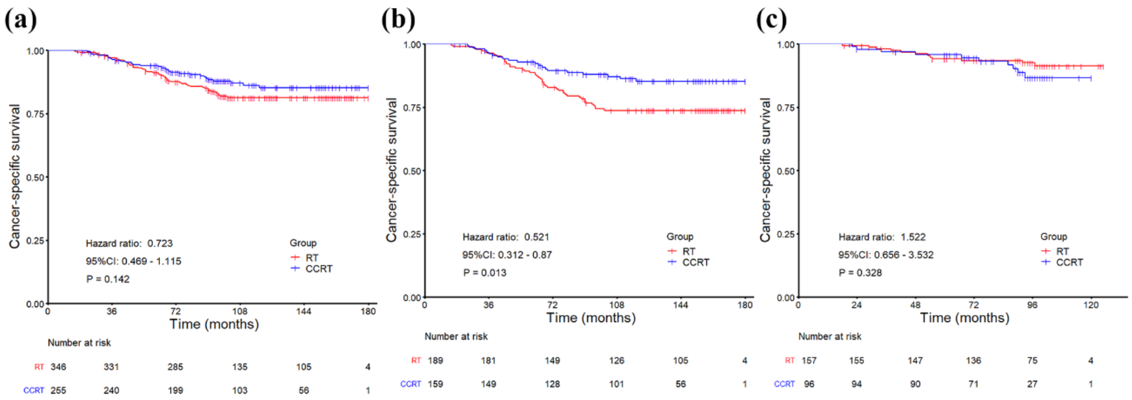

Figure S2. Kaplan-Meier survival curves for the RT and CCRT groups based on clinical stage in 2DRT subgroup: (a-e) T2N0 NPC population, (f- j) T1-2N1 NPC population. Abbreviation: RT=radiotherapy, CCRT=concurrent chemoradiotherapy, 2DRT=two-dimensional radiotherapy.

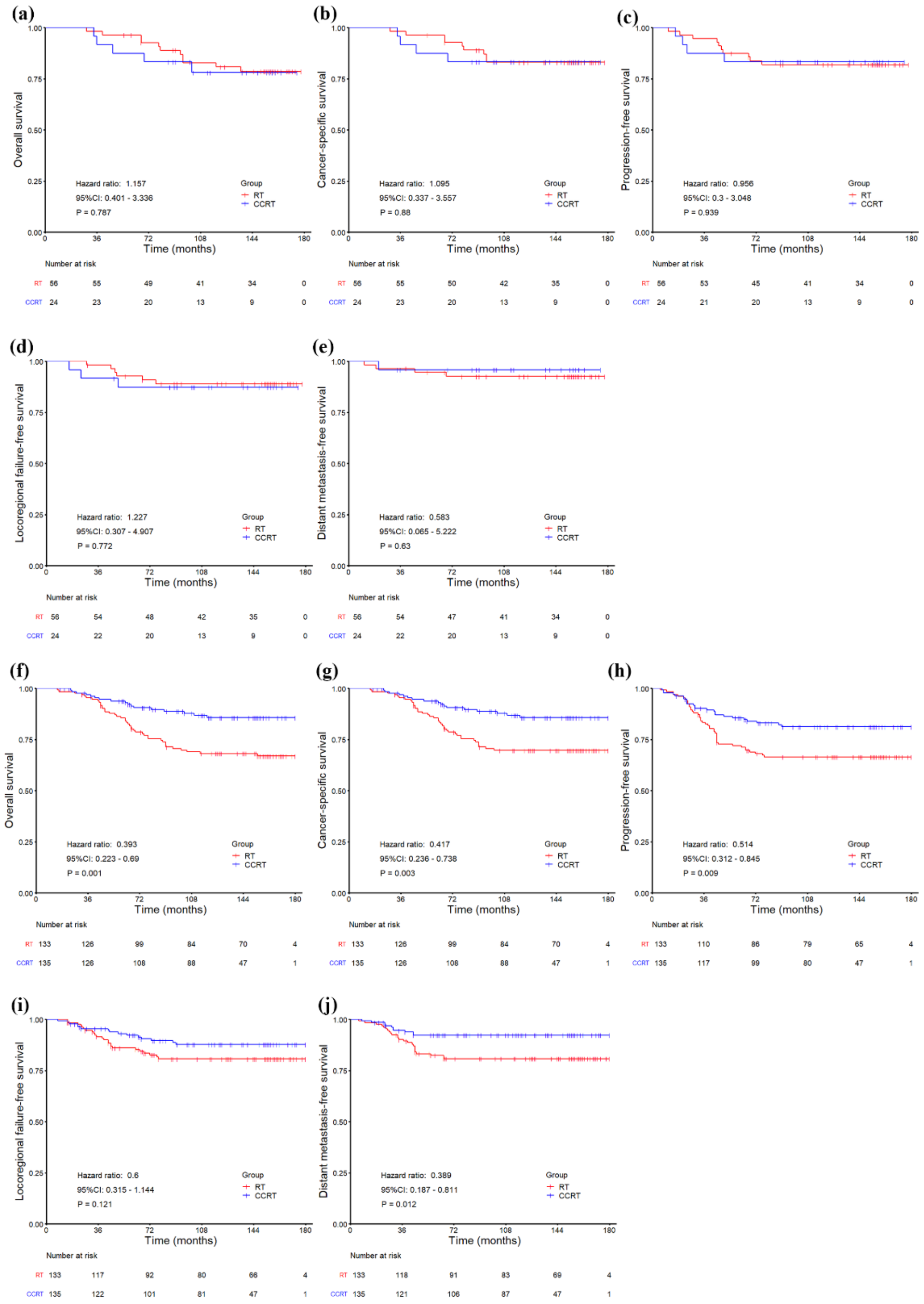

Figure S3. Kaplan-Meier survival curves for the RT and CCRT groups based on clinical stage in IMRT subgroup: (a-e) T2N0 NPC population, (f-j) T1-2N1 NPC population. Abbreviation: RT=radiotherapy, CCRT=concurrent chemoradiotherapy, IMRT=intensity-modulated radiotherapy.

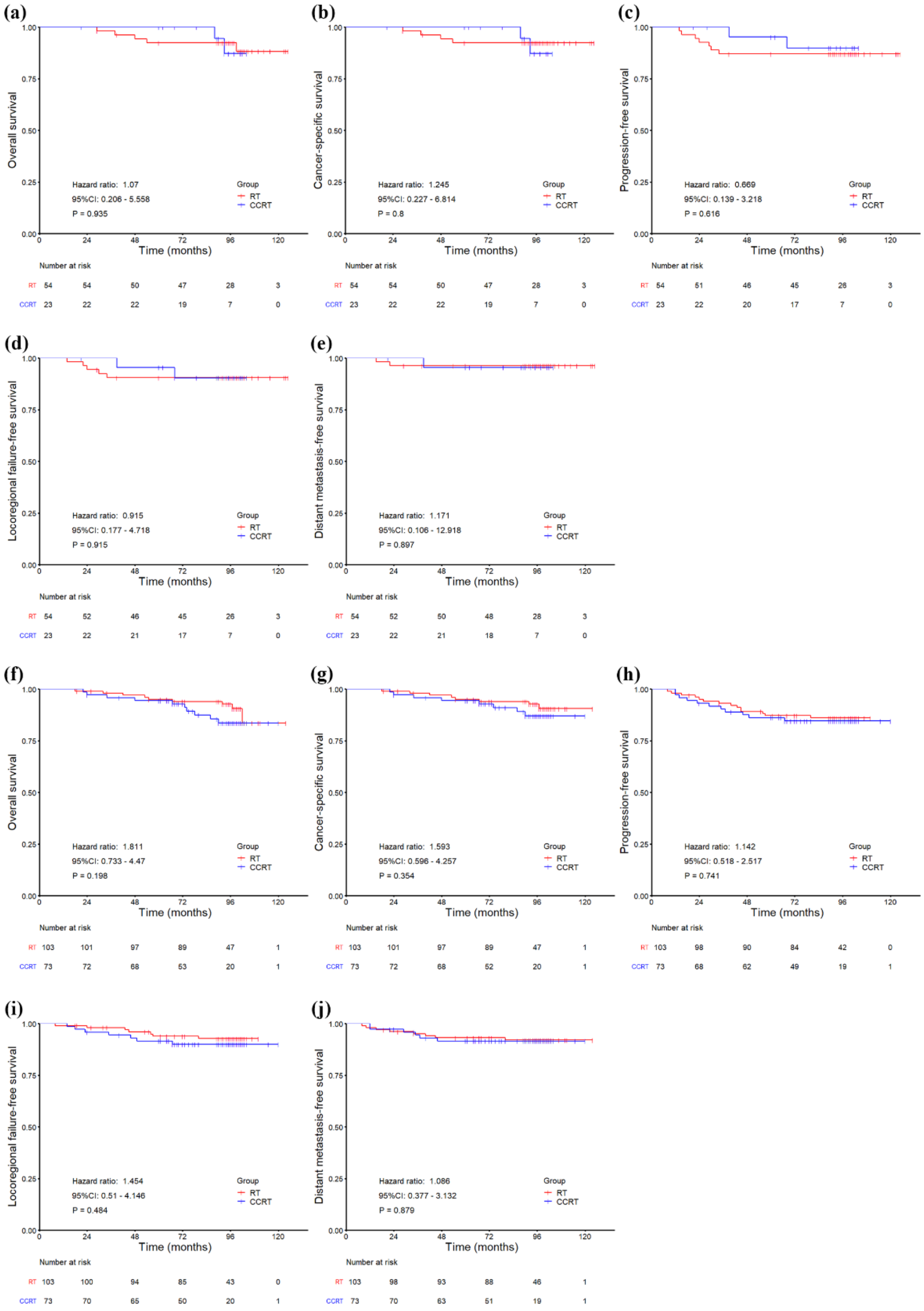

Supplement: Supplementary file 1 [file DataSheet1.pdf]
